# Views and experiences regarding workplace genetic testing: findings from a national survey of U.S. employees

**DOI:** 10.1007/s12687-025-00856-6

**Published:** 2026-03-21

**Authors:** Drew Blasco, Sarah McCain, Subhamoy Pal, Wendy R. Uhlmann, Rebecca Ferber, Kunal Sanghavi, Elizabeth Charnysh, Anya E.R. Prince, Charles Lee, J. Scott Roberts

**Affiliations:** 1 Department of Social and Behavioral Health, School of Public Health, University of Nevada, Las Vegas, Las Vegas, Nevada, USA; 2 Department of Health Behavior & Health Equity, University of Michigan School of Public Health, Ann Arbor, Michigan USA; 3 Michigan Alzheimer’s Disease Research Center, University of Michigan School of Medicine, Ann Arbor, MI USA; 4 Departments of Human Genetics and Internal Medicine, University of Michigan School of Medicine, Ann Arbor, Michigan USA; 5 Department of Internal Medicine, University of Michigan School of Medicine, Ann Arbor, Michigan USA; 6 The Jackson Laboratory for Genomic Medicine, Farmington, Connecticut USA; 7 University of Iowa College of Law, Iowa City, Iowa USA

**Keywords:** Workplace wellness programs, Workplace genetic testing (wGT), Interest, Ethical, legal, and social implications (ELSI), Employees, Testing uptake

## Abstract

The emergence of voluntary health-related genetic testing in workplace wellness programs indicates a need to understand employees’ views and experiences regarding workplace genetic testing (wGT). A large, diverse national sample of employed adults (*N*=2000; median age=43 years; 51.1% female; 33% non-white) completed a web survey of their wGT views and experiences. Although 80% of participants indicated their employer did not offer wGT, 54.1% were interested in testing, especially (somewhat/very) for cancer (89.2%), heart disease (93.1%), and Alzheimer’s disease (85.3%). Characteristics associated with wGT interest included younger age (<55 years), Hispanic/Latino ethnicity, genetic testing experience or familiarity, and a positive family medical history (all *p*<0.05). Reasons for pursuing wGT (e.g., inform health behaviors) were endorsed more frequently than reasons for declining (e.g., insurance/employability concerns). Among participants offered wGT (20%), 60% indicated having undergone testing. Test uptake was associated with personal medical history, greater familiarity with genetic testing, and self-reported physical health (all *p*<0.05). A minority of employed adults surveyed reported being offered, or having had, wGT. Nevertheless, many employees are potentially interested in wGT, particularly for common diseases. Although employees have wGT concerns, many view it as beneficial to inform health behaviors and decisions.

**Supplementary Information:**

The online version contains supplementary material available at 10.1007/s12687-025-00856-6.

## Introduction

In recent decades, genetic testing has become increasingly available with expanded access beyond clinical settings, through direct-to-consumer (DTC) companies (e.g., 23andMe) (U.S. FDA 2017) and some workplace wellness programs (Briscoe et al. 2024; Cohn et al. 2023; McDonald et al. 2020; Sanghavi et al. 2022; Singer 2018). Workplace genetic testing (wGT) is offered by employers (Deverka et al. 2020) and generally includes voluntary testing to identify risks for adult-onset conditions, including hereditary cancer and heart disease (Briscoe et al. 2024) and/or medication response (e.g., pharmacogenomics) (Charnysh et al. 2024), though the types of tests offered may vary (Cohn et al. 2023; Kroger Health 2019; McDonald et al. 2020). Employers who offer genetic testing typically do so as part of existing employer-sponsored wellness program activities (Briscoe et al. 2024; EEOC n.d.; Sanghavi et al. 2022), promoting benefits for employees (e.g., better health) and the employer (e.g., reduced healthcare costs through early risk identification and proactive actions, such as screening individuals at elevated risk) (Cohn et al. 2023; Deverka et al. 2020; McDonald et al. 2020; Sanghavi et al. 2022). There is limited information on the number of U.S. companies actually offering wGT. A recent landscape analysis found that relatively few companies were offering such testing, though methodological challenges in estimating wGT prevalence were noted (Cohn et al. 2023). This analysis also found Color Health to be the most frequent vendor of wGT utilized by employers, using a CLIA (Clinical Laboratory Improvement Amendments)-certified laboratory to generate genetic testing results for employees (Cohn et al. 2023; Color Health Inc. 2020).

The emergence of such models offered without clinician involvement (e.g., DTC, wGT) has attracted attention from scholars focused on the ELSI (i.e., ethical, legal, and social implications) of genetics due to concerns about the potential loss of confidentiality/lack of privacy (Abul-Husn et al. 2021; Brandt-Rauf et al. 2011; Briscoe et al. 2024; Clayton et al. 2018; Sanghavi et al. 2021), data sharing practices (Briscoe et al. 2023; Briscoe et al. 2024; Clayton et al. 2018; Sanghavi et al. 2021), genetic discrimination (Abul-Husn et al. 2021; Clayton et al. 2018; Geppert and Roberts 2005; Lenartz et al. 2021; Roberts et al. 2005; Roberts et al. 2011b; Willard et al. 2024), and the possibility that recipients of genetic testing may misinterpret their results (Caulfield and McGuire 2012; Marzulla et al. 2021; Petersen and Lefferts 2020; Schaper et al. 2019). wGT raises ELSI concerns similar to those of DTC, population genetic testing, and biobank use, including the privacy of genetic test results from employers and third parties (Abul-Husn et al. 2021; Caulfield and McGuire 2012; Clayton et al. 2018; Petersen and Lefferts 2020; Sanghavi et al. 2021; Sanghavi et al. 2022), the potential for employment discrimination (Clayton et al. 2018; Geppert and Roberts 2005; Roberts et al. 2005; Sanghavi et al. 2021), the inherently sensitive nature of genetic information compared to other health information (Roberts et al. 2005; Roberts et al. 2011b), and the possibility of negative psychological reactions in response to test results (Abul-Husn et al. 2021; Caulfield and McGuire 2012; Roberts 2019; Waltz et al. 2018). While such risks exist, studies have also identified potential interest in employer-offered genetic testing (Briscoe et al. 2023; Sanghavi et al. 2021), which may be driven by perceived benefits, including receiving health risk information and potentially medically actionable results (Briscoe et al. 2023; Caulfield and McGuire 2012; Charnysh et al. 2024; McDonald et al. 2020; Petersen and Lefferts 2020; Roberts et al. 2017), informing health behavior changes (Charnysh et al. 2024; McDonald et al. 2020; Roberts et al. 2017), and providing important information for one’s family (Briscoe et al. 2023; Feldman et al. 2024; Petersen and Lefferts 2020; Roberts 2019). For any genetic testing, appropriate pretest education should cover information on genetic discrimination protections (Lenartz et al. 2021; Willard et al. 2024) and whether data-sharing safeguards are in place (Briscoe et al. 2024; Caulfield and McGuire 2012; Clayton et al. 2018; Petersen and Lefferts 2020; Roberts et al. 2005) so employees can assess the risk-benefit ratio of testing (Cohn et al. 2023; Sanghavi et al. 2022).

While studies have begun to examine the landscape of wGT (Cohn et al. 2023)–including exploring employees’ behaviors following wGT at one institution (Charnysh et al. 2024), and a large survey of lay perceptions of wGT (Briscoe et al. 2024)–research is still limited, warranting further examination among employed adults to better understand the frequency and types of such testing being offered, its uptake among those who are offered it, and interest among working adults who may be offered testing in the future. While definitions of genetic testing offered through the workplace have varied, this study defines wGT as optional genetic testing programs offered by employers in addition to typical healthcare benefits. The overall goal of this study was to better understand the interest, uptake, and attitudes toward wGT among a large, diverse national sample of U.S. working adults. We specifically aimed to (a) estimate the proportion of employed adults reporting employer-offered workplace wellness activities and whether genetic testing programs were offered through the workplace (wGT); (b) understand the extent to which those with access participated in such programs; (c) understand general employee interest in wGT, including disease-specific testing (e.g., cancer, Alzheimer’s disease, heart disease); (d) understand reasons for interest or non-interest in wGT and describe participants’ preferences for wGT design features for inclusion in such testing; and (e) determine participant characteristics that may be associated with uptake or interest in wGT.

## Materials & methods

### Data collection

A survey on wGT uptake, interest, and attitudes was administered to a large national sample of participants (*N*=2000) from February to March 2022. A third-party survey firm, Dynata, was utilized for participant recruitment and survey administration. Dynata maintains a large survey panel of employed adults across the U.S. and various employer sectors and occupations. As part of survey administration, Dynata uses quality control measures to validate survey takers and identify anomalies and outliers in survey-taking behavior.

Eligible individuals were 18 years or older and employed by a third-party employer (i.e., not self-employed), full or part-time. Participants were compensated per Dynata’s existing negotiated rates. Participants reviewed an online study information sheet and provided informed consent before accessing the web survey. The Institutional Review Board at the University of Michigan approved this study as exempt (HUM00210267).

### Survey measures

The analyses utilized specific survey items (described below) that were part of a larger survey focused on the ELSI of wGT. An interdisciplinary study team in ELSI research, genetic counseling, health law, public health, and survey methods developed the survey based on a literature review and their expertise. Several members of this interdisciplinary study team were part of other studies on genetic testing, such as PGen (The Impact of Personal Genomics Study), which examined personal genomic testing experiences (Carere et al. 2014) and the REVEAL (The Risk Evaluation and Education for Alzheimer’s Disease Study) series of clinical trials on the return of *APOE* genetic test results (Roberts et al. 2011a). When designing this wGT survey, we drew upon these experiences, although we did not use verbatim survey items from those studies. Further, this current study builds on a pilot study that examined the perspectives of employees at The Jackson Laboratory (*N*=594) regarding hypothetical genetic testing, including wGT (Sanghavi et al. 2021). Survey questions relevant to this analysis are included in Supplement 1.

### Demographics & sample characteristics

Participants’ demographic information included sex, age, race, ethnicity, education, and household income. Additional questions were asked about participants’ employment sector, occupations, and whether they worked full or part-time. Participants were also asked about their physical health and personal and family history of medical conditions. Finally, questions were asked to assess participants’ familiarity with and prior genetic testing uptake outside employer-sponsored opportunities.

### Employer-offered workplace wellness activities & genetic testing programs

Participants were informed in the survey that “*Many employers now have workplace wellness activities: promotions and programs aimed at supporting healthy behavior and improving health outcomes among employees. For example, an employer might offer smoking cessation or walking step programs, free blood pressure screening, or gym membership subsidies.*” Subsequently, they were asked if their employer offered any workplace wellness activities and, if so, whether they had participated.

Similarly, participants were informed that “*Many employers are now also offering optional genetic testing programs. These programs are offered in addition to typical healthcare benefits and might include testing to identify genetic risks for common health conditions such as cancer and heart disease.*” They were then asked if their employer offered a genetic testing program (wGT) and whether they had participated. Participants are referred to as “offered wGT” and “not offered wGT” hereafter based on their response to this question. Additionally, participants offered wGT were asked what types of genetic tests were available and how testing was administered (e.g., onsite, outside genetic testing company, etc.).

### Interest in wGT

Participants not offered wGT were asked whether they would be interested if it were available. All participants were asked how interested they would be in genetic testing for medical conditions (e.g., *cancer, heart disease, Alzheimer’s disease*)*,* carrier testing, ancestry, and other types of testing [e.g., *pharmacogenomic, nutrigenetic, fitness, specific traits* (e.g., *cleft chin, alcohol flush reaction*) *testing,* and *genetic markers that predict reactions to workplace toxin exposures*].

All participants were asked a series of similar questions about decision-making factors regarding testing (Supplemental Information 1). First, all participants were asked about the importance of potential test benefits in deciding whether to undergo wGT (e.g., results informing medical care, planning for the future, informing health behaviors/lifestyle choices). Second, participants were asked about the importance of potential test risks/limitations in deciding whether to undergo wGT (e.g., results may make one worried/anxious, results may not be useful/difficult to understand, and results may impact insurance).

### wGT preferences

All participants were informed that there were several ways employers could offer wGT and asked to indicate which options (assuming their employer would pay all the associated costs) they would consider and most prefer [e.g., onsite or offsite providers, primary care providers (PCP), genetics clinics, etc.]. Participants were also asked their opinions regarding the importance of wGT design features (e.g., assurance that employees’ test results would not be shared with supervisors/coworkers, assurance that employees’ test results would not impact their health insurance, service provided at no cost to employees).

### Data analyses

Descriptive statistics were calculated regarding participants’ uptake and interest in wGT, preferences for ways it may be offered, and important wGT design features. A two-sample proportional test was conducted to determine whether there was a significant difference between participants whose employers offered wGT and those that did not in their responses regarding the importance of reasons for interest or non-interest in wGT. Two multivariable logistic regression models were conducted to examine how participant demographics and other key characteristics may be associated with wGT uptake and interest. These models examined participant demographics and other key characteristics to identify associations with (a) participants offered wGT, wherein uptake was defined as *yes* versus *no/not sure*, and (b) participants not offered wGT, wherein interest was defined as *definitely/probably yes* versus *not sure/probably not/definitely not*. The same demographic and characteristic variables were included in each regression: sex (female, male); age (18–34, 35–54, 55 and above); race and ethnicity (Asian, Black, non-Hispanic, Hispanic/Latino, Other, white, non-Hispanic); education (professional degree, college graduate, some college, high school or less); household income ($100,000 and more, $50,000-$99,999, less than $50,000); employment sector (healthcare/pharmaceutical, technology/biotechnology, other); familiarity with genetic testing (very familiar, somewhat familiar, not very familiar); self-reported physical health (excellent/very good, good, fair/poor); personal history of medical conditions (one or more, none); family history of medical conditions (one or more, none); and prior genetic testing uptake (yes, no). Data analysis was conducted in R Studio version 2024.12.1+563 using R version 4.2.3.

## Results

### Participant characteristics

Of the 2000 participants included in our analysis, the median age was 43 years, and most had completed postsecondary education (55.1% college graduate/professional degree). Participants were roughly evenly split by sex (51.1% female), and the sample was racially/ethnically diverse (13.6% Black, non-Hispanic, 8.4% Hispanic/Latino, and 8.1% Asian) (Table 1). Almost one-fourth (23.7%) of participants reported being in the healthcare, pharmaceutical, technology, or biotechnology sectors. Participants’ occupations were highly diverse, including representation from all categories reported by the U.S. Bureau of Labor Statistics (2024) from the “Occupational Employment and Wage Statistics” with the top three categories in our sample including: “Education, training, and library” (11.1%); “Sales” (9.6%); and “Management” (9.3%).

**Table 1:** Sample Characteristics (*N*=2000)

Variables	N (%)
*Sex:*	
Female	1022 (51.1)
Male	978 (48.9)
*Age*^*a*^*:*	
18–34	636 (31.8)
35–54	858 (42.9)
55 and above	505 (25.3)
*Race & Ethnicity:*	
Asian	163 (8.1)
Black, non-Hispanic	272 (13.6)
Hispanic/Latino	167 (8.4)
Other^*b*^	54 (2.7)
White, non-Hispanic	1344 (67.2)
*Education:*	
High School or Less	292 (14.6)
Some College	606 (30.3)
College Graduate	663 (33.1)
Professional Degree	439 (22.0)
*Household Income:*	
Less than $50,000	479 (24.0)
$50,000-$99,999	838 (41.9)
$100,000 or more	683 (34.1)
*Employment Sector:*	
Healthcare/Pharmaceutical	272 (13.6)
Technology/Biotechnology	203 (10.1)
Other	1525 (76.3)
*Familiarity with Genetic Testing:*	
Very Familiar	326 (16.3)
Somewhat Familiar	1127 (56.4)
Not Very Familiar	547 (27.3)
*Prior Genetic Testing Uptake:*	
Yes^*c*^	918 (45.9)
Healthcare Provider	515 (25.8)
DTC Service	429 (21.5)
Research Study	124 (6.2)
Two or more Types of Genetic Testing	131 (14.3)
No	1082 (54.1)
*Self-Reported Physical Health:*	
Fair/Poor	207 (10.4)
Good	652 (32.6)
Excellent/Very Good	1141 (57.0)
*Personal History of Medical Conditions*^*d*^*:*	
One Disease	559 (27.9)
Two or More Diseases	206 (10.3)
None	1235 (61.8)
*Family History of Medical Conditions*^*d*^*:*	
One Disease	454 (22.7)
Two or More Diseases	944 (47.2)
None	602 (30.1)

^*a*^Age: *n*=1999 due to missing data.

^*b*^Other Category: Includes participants identifying as (race): *American Indian or Native Alaskan, Native Hawaiian or other Pacific Islander, Multi-race, none of these describe me;* (ethnicity): *Middle Eastern/North African, Multi-ethnic, none of these describe me.*

^*c*^Prior Genetic Testing: Overall (in general) “yes” to having prior genetic testing, which could include one or more of the following: through a *healthcare provider, DTC service*, and/or a *research study.*

^*d*^Medical Conditions: *Cancer, diabetes, drug addiction/substance abuse disorder, eye condition, heart disease, neurological condition, psychiatric condition, genetic condition*.

Due to rounding, percentages may not add up to 100%.

Abbreviations: N (Total Number); DTC (Direct-to-Consumer).

Most participants reported being familiar (*somewhat/very*) with genetic testing (72.7%), among which 45.9% reported having had prior genetic testing completed through one or more of the following ways: a healthcare provider (25.8%), DTC service (21.5%), or a research study (6.2%). Only 14.3% of participants had two or more types of prior genetic testing. Most participants reported being healthy, with only 10.4% indicating health as fair/poor. Most participants (69.9%) reported a family history of at least one medical condition, with diabetes (39.7%), cancer (39.5%), heart disease (30.9%), and psychiatric conditions (20.3%) most frequently reported. A personal history of disease was reported by 38.2% of participants, with a psychiatric condition (15.4%), diabetes (10.9%), eye condition (7.6%), and heart disease (5.3%) being the most common.

### Prevalence & uptake of employer-offered workplace wellness activities or wGT

More than half of the participants (54.4%) reported their employer offered workplace wellness activities, with most (80%) indicating that their employer either did not offer or they were unsure if their employer offered wGT within workplace wellness activities (Table 2). Of those offered wGT (20%), 60.3% reported they had participated. Among all participants who were surveyed, few (4.5%) reported they had not participated in wGT and that they would not do so in the future (Supplemental Figure 1). The most commonly reported way wGT was offered was through an onsite service provided by their employer (41.5%) and testing through an outside genetic testing company chosen by their employer (33.0%). Testing through one’s PCP (15.8%), through a genetics clinic (5.0%), and through a participant-chosen genetics company (3.8%) were less commonly endorsed. Close to or greater than 40% of participants reported being offered genetic testing for fitness testing and heart disease/cancer risk. Roughly 30% reported carrier testing and genetic markers that predict reactions to workplace toxin exposures (e.g., Beryllium), and more than 20% reported testing to predict medication response (i.e., pharmacogenomic testing) and nutrigenetic testing. Less commonly reported genetic tests were ancestry testing and testing for certain traits (e.g., alcohol flush reaction, cleft chin) (both 17.0%) and risk of other medical conditions (e.g., diabetes, 1.2%).

**Table 2:** Employer Offered Programs: Uptake & Interest

***Employer Program Offered (N=2000)***			
**Program**	**Yes N (%)**	**No N (%)**	**Not Sure N (%)**
Workplace Wellness Activities	1088 (54.4)	778 (38.9)	134 (6.7)
Workplace Genetic Testing (wGT)	400 (20.0)	1292 (64.6)	308 (15.4)
***Employer Program Uptake (N=2000)***			
**Program**	**Yes N (%)**	**No, but may in the future N (%)**	**No, and don’t plan to in the future N (%)**
Workplace Wellness Activities (*n*=1088)	738 (67.8)	302 (27.8)	48 (4.4)
Workplace Genetic Testing (wGT) (*n*=400)	241 (60.3)	141 (35.3)	18 (4.5)
***Way in which Workplace Genetic Testing Offered (n=400)***			
	**N (%)**		
Onsite service provided by employer	166 (41.5)		
Outside genetic testing company chosen by my employer	132 (33.0)		
Own PCP	63 (15.8)		
Genetics clinic	20 (5.0)		
Genetic company that individual chooses	15 (3.8)		
Other	4 (1.0)		
***Genetic Testing Types (n=241)***			
**Type of Genetic Test**	**N (%)**		
Cancer Risk	111 (46.1)		
Fitness Testing (to inform exercise regimen)	106 (44.0)		
Heart Disease Risk	95 (39.4)		
Reactions to Workplace Toxin Exposure (e.g., Beryllium)	71 (29.5)		
Carrier Testing	70 (29.0)		
Pharmacogenomics (response to medications)	63 (26.1)		
Nutrigenetic Testing (to inform response to food/diet)	50 (20.7)		
Ancestry Testing	41 (17.0)		
Testing for Certain Traits (e.g., alcohol flush reaction)	41 (17.0)		
Risk of Other Medical Conditions	3 (1.2)		
Other	3 (1.2)		
Don’t Know	9 (3.7)		

Abbreviations: N (Total Number); PCP (Primary Care Provider); wGT (Workplace Genetic Testing).

### Interest in wGT & reasons for testing

For participants not offered wGT (*n*=1600), slightly over half (54.1%) reported being probably or definitely interested in such testing, with few endorsing being probably (9.9%) or definitely (6.9%) uninterested if offered (Table 3). Among the entire sample, participants demonstrated at least some interest in all test types, with the highest interest (somewhat/very: >80%) in risk for heart disease, cancer, Alzheimer’s disease, and nutrigenetic testing; about 70% or more reported interest in ancestry, fitness, carrier, pharmacogenomics, workplace toxin exposures, and trait testing.

**Table 3:** Interest in & Preferences for Workplace Genetic Testing

N (%)					
	***Definitely Yes***	***Probably Yes***	***Not Sure***	***Probably Not***	***Definitely Not***
***Health-Related Genetic Test Interest*** (*n*=1600)	312 (19.5)	554 (34.6)	465 (29.1)	158 (9.9)	111 (6.9)
***Interest in Specific Genetic Tests*** (*N*=2000)	***Not at all***	***Somewhat***		***Very***	
Heart Disease	212 (10.6)	715 (35.8)		1073 (57.3)	
Cancer	218 (10.9)	711 (35.6)		1071 (53.6)	
Alzheimer’s Disease	295 (14.8)	717 (35.9)		988 (49.4)	
Ancestry Testing	418 (20.9)	666 (33.3)		916 (45.8)	
Nutrigenetic Testing	388 (19.4)	789 (39.5)		823 (41.1)	
Fitness Testing	442 (22.1)	782 (39.1)		776 (38.8)	
Carrier Testing	528 (26.4)	704 (35.2)		768 (38.4)	
Pharmacogenomics	409 (20.5)	904 (45.2)		687 (34.4)	
Reactions to Workplace Toxin Exposure	537 (26.9)	796 (39.8)		667 (33.3)	
Testing for Certain Traits	615 (30.8)	747 (37.4)		638 (31.9)	
***Importance of Workplace Genetic Testing Design Features***	***Not at all***	***Somewhat***		***Very***	***Extremely***
Assurance that test results won’t impact health insurance	70 (3.5)	278 (13.9)		451 (22.6)	1201 (60.1)
Assurance that employees’ test results will not be shared with supervisors/co-workers	96 (4.8)	320 (16.0)		429 (21.5)	1155 (57.8)
Data not shared without consent of employees, even if de-identified	92 (4.6)	300 (15.0)		488 (24.4)	1120 (56.0)
Provision of genetic counseling services to help interpret results	121 (6.1)	499 (25.0)		747 (37.4)	633 (31.7)
No cost testing	91 (4.6)	295 (14.8)		510 (25.5)	1104 (55.2)
Testing done at work site for convenience	353 (17.7)	630 (31.5)		532 (26.6)	485 (24.3)
Information provided about laws that protect against genetic discrimination	106 (5.3)	393 (19.7)		650 (32.5)	851 (42.6)
***Ways Employers May Offer Testing***^***a***^	***N (%)***			***Ranking: Most Preferred (n, %)***^***b***^	
Through own PCP	1057 (52.9)			1: (730, 36.5)	
Through onsite service provided by employer	714 (35.7)			2: (447, 22.4)	
Through outside genetic testing company chosen by employer	707 (35.4)			3: (321, 16.1)	
Through genetic company that individual chooses	606 (30.3)			4: (314, 15.7)	
Through genetics clinic	543 (27.2)			5: (188, 9.4)	

^*a*^Participants were told to assume that under each option, their employer would pay all costs associated with the testing.

^*b*^Ranking of methods from most (1) to least (5) preferred.

Abbreviations: N (Total Number); PCP (Primary Care Physician).

### Importance of reasons for interest or non-interest in wGT

**Fig 1:**
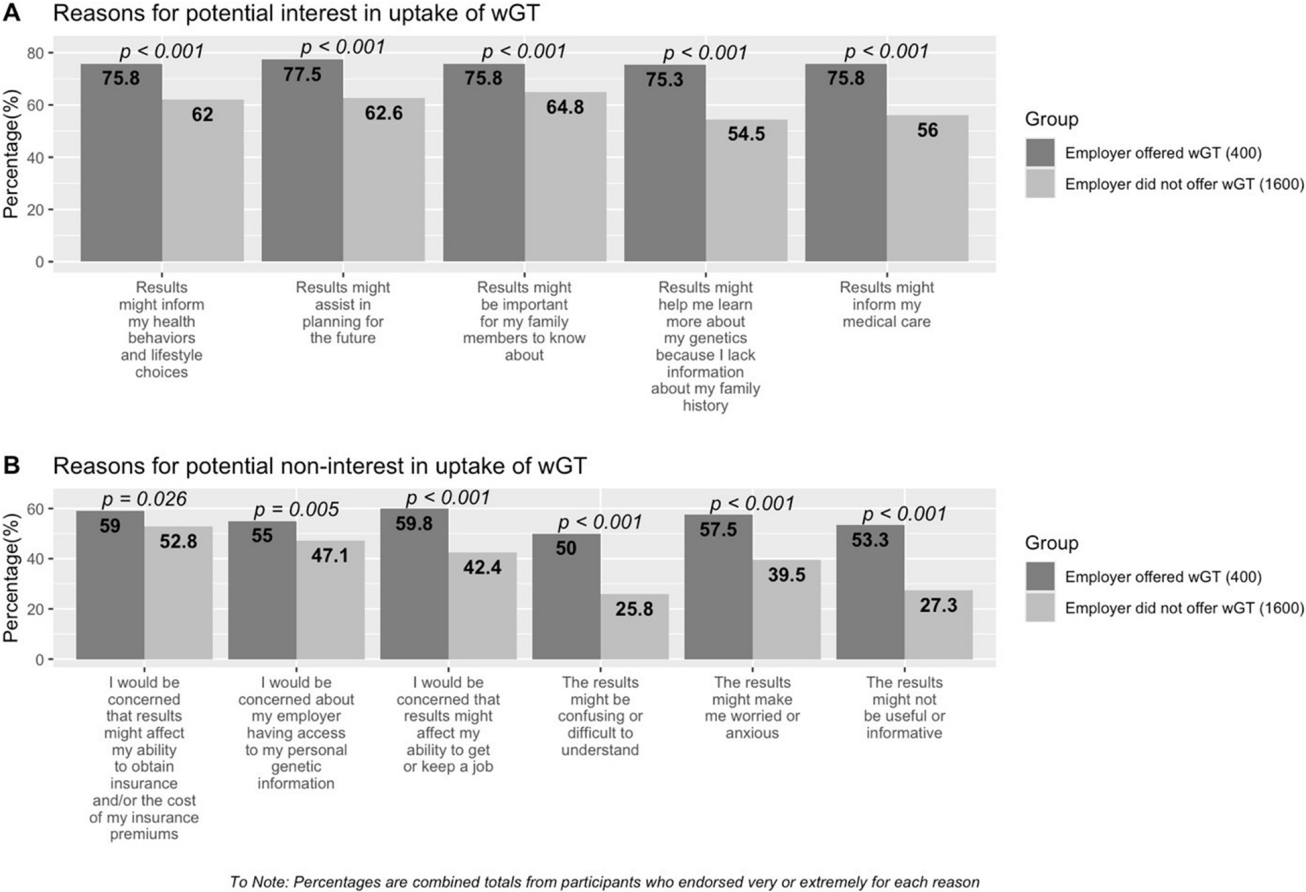
Combined percentages for participants reporting *very/extremely important* for (**A**) reasons for potential interest and (**B**) reasons for potential non-interest in the uptake of workplace genetic testing

Among participants offered wGT (*n*=400), more than 75% indicated the following as very/extremely important reasons for seeking testing: informing medical care, health behaviors, and lifestyle choice; assisting in future planning; the importance of results for family members; and learning about one’s genetics due to a lack of family history (Fig. 1). Few participants reported these reasons as not at all important to their test decision-making (≤8% for all reasons).

Among participants not offered wGT, combined percentages (very/extremely important) were somewhat lower compared to those offered wGT, with the following items endorsed greater than 60% by participants: results might assist in planning for the future; informing my health behaviors and lifestyle choices; and might be important for my family members. Compared to those offered wGT, more participants not offered wGT reported “not at all” in response to the importance of these reasons (<15% among all reasons).

Half or more of the participants offered wGT reported the following reasons as very/extremely important for potential non-interest in wGT: results might not be useful/informative (53.3%); make them worried/anxious (57.5%); be confusing/difficult to understand (50%); concerns about employer’s access to genetic information (55%); the impact on obtaining insurance and/or the cost of insurance premiums (59%); and the ability to get/keep a job (59.8%). A smaller proportion of participants reported “not at all” for these items (range: 15.8-22.3%).

Among participants not offered wGT, combined percentages of very/extremely important were as follows for the potential reasons for non-interest in wGT: concern about results impacting obtaining insurance/its cost (52.8%); concern about employer access to genetic information (47.1%); affecting my ability to get/keep a job (42.4%); make me worried/anxious (39.5%); results might not be useful/informative (27.3%); and might be confusing/difficult to understand (25.8%). Overall, participants more frequently endorsed “not at all” in this group compared to those offered wGT (range: 16.0-32.6%). Participants offered wGT by their employer had a significantly higher endorsement of both reasons for potential interest and non-interest in uptake of wGT compared to those participants who indicated that their employer did not offer wGT (*p*<0.05 for all reasons).

### Preferences for wGT features

When asked to select all the genetic testing methods they would consider, participants selected testing through their PCP (52.9%), an onsite service (35.7%), an outside company provided by their employer (35.4%), any genetic testing company of their choice (30.3%), and a genetics clinic (27.2%) (Table 3). When participants were asked about their most preferred option, the most highly ranked was testing via their PCP, with the least being testing via a genetics clinic.

The top reported (very/extremely important) wGT features were assurance that their test result would not impact their health insurance (82.7%), no-cost testing (80.7%), and data would not be shared without employee permission, even if de-identified (80.4%). All features were endorsed by more than 50% of participants as very/extremely important (Table 3).

### Factors associated with wGT uptake

Among participants offered wGT, demographic characteristics (i.e., sex, age, race and ethnicity, education, household income, employment sector) were not associated with wGT uptake. However, familiarity with such testing, a personal history of at least one medical condition, and self-reported physical health were associated with wGT uptake (Table 4). More specifically, participants who reported being very familiar with genetic testing were more likely to have participated in wGT compared to those not very familiar [adjusted odds ratio (aOR)=4.25; 95% CI: 1.59, 11.9]. Participants who reported having at least one medical condition were more likely to have participated in wGT compared to those who reported no medical conditions (aOR=1.97; 95% CI: 1.11, 3.52). Finally, participants who reported good physical health were less likely to have participated in wGT compared to those who reported excellent/very good physical health (aOR=0.39; 95% CI: 0.22, 0.69).

**Table 4:** Logistic Regressions for Workplace Genetic Testing (wGT) Uptake & Interest

	‘Offered wGT’ Uptake (*N*=400)			‘Not Offered wGT’ Interest (*N*=1599)		
Variable	aOR	(95% CI)	*P*-Value	aOR	(95% CI)	*P*-Value
*Sex:*						
Male	0.75	(0.45, 1.26)	0.28	1.18	(0.95, 1.46)	0.14
Female	1.00			1.00		
*Age:*						
18–34	0.53	(0.21, 1.31)	0.18	**2.03**	**(1.52, 2.72)**	**<0.001**
35–54	0.51	(0.20, 1.26)	0.15	**1.58**	**(1.23, 2.03)**	**<0.001**
55 and above	1.00			1.00		
*Race & Ethnicity:*						
Asian	0.85	(0.31, 2.39)	0.76	1.16	(0.80, 1.69)	0.45
Black, non-Hispanic	1.24	(0.64, 2.42)	0.52	1.08	(0.78, 1.52)	0.63
Hispanic/Latino	1.77	(0.86, 3.73)	0.13	**1.68**	**(1.10, 2.62)**	**0.019**
Other^*a*^	0.60	(0.11, 2.91)	0.54	0.92	(0.48, 1.75)	0.79
White, non-Hispanic	1.00			1.00		
*Education:*						
High School or Less	1.25	(0.66, 2.38)	0.72	1.10	(0.75, 1.62)	0.64
Some College	0.63	(0.31, 1.27)	0.19	1.07	(0.77, 1.47)	0.70
College Graduate	0.85	(0.34, 2.11)	0.49	1.26	(0.93, 1.71)	0.13
Professional Degree	1.00			1.00		
*Household Income:*						
Less than $50,000	0.66	(0.31, 1.41)	0.28	0.76	(0.56, 1.04)	0.09
$50,000-$99,999	0.68	(0.38, 1.20)	0.18	0.84	(0.65, 1.08)	0.17
$100,000 and more	1.00			1.00		
*Employment Sector:*						
Healthcare/Pharmaceutical	0.62	(0.31, 1.25)	0.18	1.19	(0.88, 1.62)	0.26
Technology/Biotechnology	0.66	(0.37, 1.16)	0.15	1.00	(0.66, 1.53)	0.99
Other	1.00			1.00		
*Familiarity with Genetic Testing:*						
Very Familiar	**4.25**	**(1.59, 11.9)**	**0.004**	**2.17**	**(1.40, 3.40)**	**<0.001**
Somewhat Familiar	1.53	(0.60, 4.08)	0.38	**1.51**	**(1.20,1.91)**	**<0.001**
Not Very Familiar	1.00			1.00		
*Prior Genetic Testing Uptake:*						
Yes	1.97	(0.99, 4.01)	0.06	**1.50**	**(1.19, 1.89)**	**<0.001**
No	1.00			1.00		
*Self-Reported Physical Health:*						
Fair/Poor	0.56	(0.15, 1.95)	0.37	1.03	(0.73, 1.46)	0.86
Good	**0.39**	**(0.22, 0.69)**	**0.001**	0.84	(0.67, 1.05)	0.13
Excellent/Very Good	1.00			1.00		
***Personal History of Medical Conditions***^*b*^*:*						
One or more diseases	**1.97**	(1.11, 3.52)	0.020	1.24	(0.98, 1.56)	0.07
None	1.00			1.00		
***Family History of Medical Conditions***^*b*^*:*						
One or more diseases	1.02	(0.58, 1.79)	0.94	**1.46**	**(1.14, 1.87)**	**0.003**
None	1.00			1.00		

^*a*^Other Category: Includes participants identifying as: (race): *American Indian or Native Alaskan, Native Hawaiian or other Pacific Islander, Multi-race, none of these describe me;* (ethnicity): *Middle Eastern/North African, Multi-ethnic, none of these describe me.*

^*b*^Medical Conditions: *Cancer, diabetes, drug addiction/substance abuse disorder, eye condition, heart disease, neurological condition, psychiatric condition, genetic condition.*

*Bolded values indicate statistical significance (*p*<0.05).

Abbreviations: wGT (Workplace Genetic Testing); N (Total Number); aOR (Adjusted Odds Ratio); CI (Confidence Interval).

### Factors associated with wGT interest

Among participants not offered wGT, age, and race and ethnicity were the only demographic characteristics associated with interest in such testing. Younger participants were more likely to be interested than older participants. Specifically, participants aged 18–34 (aOR=2.03; 95% CI: 1.52, 2.72) and those aged 35–54 (aOR=1.58; 95% CI: 1.23, 2.03) were more interested in wGT compared to those aged 55 and above. Participants who identified as Hispanic/Latino were more likely to be interested (aOR=1.68; 95% CI: 1.10, 2.62) compared to non-Hispanic, white participants.

Similarly, familiarity with genetic testing, prior testing uptake, and a family history of medical conditions were associated with wGT interest. Participants who reported being very (aOR=2.17; 95% CI: 1.40, 3.40) and somewhat familiar (aOR=1.51; 95% CI: 1.20, 1.91) with genetic testing were more likely to be interested in participating in wGT compared to those who were not very familiar. Participants who reported prior uptake of genetic testing (outside the workplace) were more likely to be interested in participating in wGT (aOR=1.50; 95% CI: 1.19, 1.89) compared to those who did not report prior testing. Finally, participants who reported having a family history of at least one medical condition were more likely to be interested in participating in wGT compared to those who reported no family history (aOR=1.46; 95% CI: 1.14, 1.87).

## Discussion

Our large, national study of employees from diverse occupations, incomes, and educational attainment contributes to the limited literature on wGT. A recent study found that relatively few U.S. employers offered wGT, aligning with this study’s findings, wherein most participants reported they were not offered wGT (Cohn et al. 2023). However, for participants offered wGT, the vast majority reported either having already participated or being open to potential future participation. While the broader ELSI community has noted concerns (e.g., privacy, genetic discrimination) about genetic testing offered outside of a clinical environment, in general, this current sample of employed adults was generally open to wGT participation, even if they had not previously completed such testing.

Prior research has noted high interest in genetic testing participation if given the opportunity among a variety of samples (e.g., a representative sample of employed U.S. adults, nationally representative sample of older adults, U.S. consumers with employer-sponsored health insurance, and employees of high-tech, large companies) (Briscoe et al. 2023; Briscoe et al. 2024; Feldman et al. 2024; Wamberg Genomic Advisors 2017); however, uptake of such testing has varied (Deverka et al. 2020; Feldman et al. 2024; Singer 2018). Results from the large Health Information National Trends Survey (HINTS) found that only 28.7% of adults reported having engaged in genetic testing, though the vast majority (81.6%) were aware of genetic testing opportunities (Memon et al. 2025). Similar to previous research, in this study, many participants reported interest in wGT if their employer offered it (Briscoe et al. 2023; Briscoe et al. 2024) and high interest in several disease-specific tests, including testing for the risk of relatively common adult-onset conditions such as cancer, heart disease, and Alzheimer’s disease (Feldman et al. 2024; Roberts et al. 2017). A recent paper explored employees’ reactions to receiving genetic testing results via wGT, including participants’ engagement with pretest education materials and its facilitation of the decision-making process, finding limited utilization of pretest education among wGT participants (Charnysh et al. 2025a). Prior research has noted that what and how much information is disclosed during the informed consent process on the Genetic Information Nondiscrimination Act (GINA) and its known gaps impact participants’ hypothetical research participation (Prince et al. 2022). Our findings were consistent with prior studies on predictive and/or DTC genetic testing in that uptake and interest in wGT were higher among those familiar with genetic testing (Ruhl et al. 2019) or those who reported prior genetic testing uptake (Briscoe et al. 2024; Ruehl et al. 2023), and those with personal/family history of medical conditions (Kaufman et al. 2012; Meisel et al. 2015). Our finding that interest in wGT was higher among Hispanic/Latino individuals was contrary to most past findings (Briscoe et al. 2024; Clayton et al. 2018; Thompson et al. 2003) but consistent with a recent study’s findings that Hispanic/Latino participants in a genomic biobank were more likely to want to receive their genomic testing results (Abul-Husn et al. 2021). These mixed findings suggest a need to consider the specific context of genetic testing when considering racial and ethnic differences in test interest and to recognize that hypothetical interest in genetic testing, as expressed in surveys, often does not translate to actual test uptake in practice.

Common issues in historical and current literature (and ELSI research) have focused on the insurance implications of genetic testing and screening, such as genetic discrimination (Abul-Husn et al. 2021; Briscoe et al. 2023; Clayton et al. 2018; Geppert and Roberts 2005; Lenartz et al. 2021; Waltz et al. 2018; Willard et al. 2024) and privacy and sharing of results, even if de-identified (Abul-Husn et al. 2021; Caulfield and McGuire 2012; Clayton et al. 2018; Geppert and Roberts 2005; Lenartz et al. 2021; Sanghavi et al. 2021; Sanghavi et al. 2022). Participants in our study similarly noted the importance of these concerns when asked about wGT design features (e.g., test results will not impact insurance, test results will not be shared with supervisors/co-workers). Additionally, participants were most likely to prefer having their PCP complete testing, with less preference for other options, such as an outside genetic testing company or a genetics clinic (Briscoe et al. 2023; Briscoe et al. 2024; Schaper et al. 2019). This finding is consistent with those of Briscoe and colleagues, who found that among a large sample of employed adults, more than half were likely to participate in genetic testing through their healthcare provider, while less than half indicated they would participate in wGT (Briscoe et al. 2024). While our participants’ preferred method of wGT was through their PCP, it is unlikely that this genetic testing model could feasibly be offered in the near future given numerous barriers, including the lack of genetics training that PCPs receive, their lack of comfort in counseling patients about genetic testing, and their lack of time to spend with individual patients on genetic testing, given competing demands (Chambers et al. 2015; Mikat-Stevens et al. 2014). Significant concerns have been raised around genetic testing models outside of a clinical setting (e.g., those offered via third-party and DTC genetic testing companies) regarding the provision of informed consent and sufficient pretest education (Deverka et al. 2020; Kilbride et al. 2018; Roberts et al. 2017; Roberts 2019; Seaver et al. 2022), privacy of results (Allyn 2024; Caulfield and McGuire 2012; Clayton et al. 2018; Geppert and Roberts 2005; Sanghavi et al. 2021; Sanghavi et al. 2022; Seaver et al. 2022), and data usage (Briscoe et al. 2024; Petersen and Lefferts 2020; Schaper et al. 2019; Seaver et al. 2022). Prior wGT research found greater intent to participate in such testing if key design features were included, such as data sharing limitations, data usage control, options to delete data, and sufficient legal protections (Briscoe et al. 2024). These design features are important to consider among stakeholders implementing genetic testing outside traditional clinical testing settings.

While ongoing commentary in this area has emphasized potential harms of testing offered outside of a clinic, often without licensed providers and/or specialists (e.g., genetic counselors), our sample of potential future users indicated high interest in such testing, especially for relatively common adult-onset conditions. As previous studies on genetic testing (e.g., clinical, DTC, wGT) have found, participants in our study commonly endorsed potential wGT benefits, including informing medical care and health information (Akinleye et al. 2011; Briscoe et al. 2023; Caulfield and McGuire 2012; Charnysh et al. 2024; Feldman et al. 2024; McDonald et al. 2020; Petersen and Lefferts 2020; Roberts et al. 2017), healthy behaviors/lifestyle choices (Charnysh et al. 2024; McDonald et al. 2020; Roberts et al. 2017), assisting in planning for the future (Akinleye et al. 2011), and the potential importance of results for family members (Abul-Husn et al. 2021; Briscoe et al. 2023; Petersen and Lefferts 2020; Waltz et al. 2018). Although participants viewed wGT as having numerous potential benefits, many also endorsed reasons for not seeking wGT. Such reasons reflect concerns reported in the broader literature on genetic testing, including results not being useful/difficult to understand (Marzulla et al. 2021; Roberts et al. 2017), negative psychological responses to results (Abul-Husn et al. 2021; Caulfield and McGuire 2012; Kilbride et al. 2018; Petersen and Lefferts 2020; Roberts 2019), concerns about how results might impact insurance and/or one’s job (Clayton et al. 2018; Lenartz et al. 2021; Roberts et al. 2005; Roberts et al. 2011b; Sanghavi et al. 2021), and who has access to one’s results, including employers (Clayton et al. 2018; Roberts et al. 2011a, 2011b; Roberts et al. 2025; Sanghavi et al. 2021; Waltz et al. 2018).

Employees’ concerns about genetic privacy have been echoed by ELSI scholars, warning against the potential for genetic discrimination in the context of wGT (Sanghavi et al. 2022). This concern was also demonstrated in a recent related study of ours (using the same survey dataset) which found that many employed adults expressed concern that their employer would not protect the privacy of their genetic/health information, especially those who are older (≥55 years), identifying as Black, non-Hispanic, or Asian, non-Hispanic, and with a family history of common diseases (Roberts et al. 2025). While privacy concerns among employees exist, results of this current analysis demonstrated that overall, reasons for wGT interest were endorsed as important more frequently than reasons not to seek wGT; this finding may help to explain the relatively high rates of test uptake observed among those offered wGT and the high interest in future testing among those not offered wGT. Further, results from a study of employees of a U.S. healthcare system who had engaged in wGT found relatively minimal adverse psychosocial impact (Charnysh et al. 2025a). These findings suggest that, consistent with prior genetic testing studies (e.g., Roberts 2019), most participants of genetic testing offered through the workplace will not experience negative psychological impacts.

Our findings may be relevant to other types of genetic testing with similar benefits and risks. For example, population genomic screening similarly assesses unselected populations (e.g., asymptomatic individuals without a relevant family history) for increased risk of disease based on one’s genetics through programs that may partner with similar genetic testing companies (e.g., Color Health, Inc.) (Foss et al. 2022). While population genomic screening pilot programs are on the rise, best practices for implementation, including design features such as who offers the testing, who returns results, etc., may change as programs expand, especially given workforce capacity issues (e.g., the lack of genetic counselors in clinical practice). A recent article examining characteristics of existing population genomic screening programs found considerable differences in the types of genes tested, from CDC Tier 1 conditions only, to inclusion of genes from the American College of Medical Genetics and Genomics secondary findings list, to whole-genome sequencing (Foss et al. 2022). Our study indicated participants were interested in a broad set of conditions, including adult-onset conditions such as Alzheimer’s disease, which is currently offered through DTC testing companies (e.g., 23andMe) (Foss et al. 2022).

This study has several strengths. Few studies have examined employees' perceptions regarding wGT (Briscoe et al. 2024), which is an evolving area. This study presents results on the uptake of, interest in, and attitudes toward wGT from a large, diverse national sample of working adults—key stakeholders whose perspectives are critical to include in this emerging area to develop a well-rounded understanding of the ELSI of wGT. However, these results should be considered in light of limitations. We found a relatively high uptake of wGT compared to prior published estimates (Cohn et al. 2023). Although we defined wGT in the survey for participants before asking follow-up questions, participants may have overinterpreted this question, resulting in an overestimate of wGT’s prevalence. Further, due to the nature of the survey content, the investigators determined it was important to define concepts such as workplace wellness programs and wGT, which align with other prior research in this area (Briscoe et al. 2024; Sanghavi et al. 2021). However, our lack of potential wGT risks in our initial survey prompt may have inadvertently contributed to bias in participants’ responses. Additionally, participants may not have been highly knowledgeable about all types of genetic testing asked about in the survey. However, the survey would have likely been too cumbersome if we had included an extensive definition for each term. Therefore, as with self-report surveys, there is a possibility that participants may have misunderstood terminology, leading to response bias. In addition, not all survey measures had been formally validated, and the survey sample was biased toward college-educated employees, thereby constraining the generalizability of findings.

This analysis contributes to the literature on employee perspectives of wGT among a large, diverse national sample. wGT provides a new avenue for genetic testing through employment. While our findings indicate employee interest in wGT, concerns must be addressed through the implementation of key design features for benefits to be fully realized. Future research should explore in-depth employee experiences related to wGT (e.g., through qualitative interviews). Other research emerging from this research project has focused on providing an in-depth perspective from genetic counselors on their experiences with wGT (Charnysh et al. 2025b). This research should prioritize further exploration of reasons for interest or non-interest in wGT, including the experiences of participants who have already participated in wGT and why they participated. This research may inform future best practices for wGT and other testing avenues for unselected populations [e.g., population genomic screening (Foss et al. 2022)]. Finally, our analyses indicated differences in interest in wGT (if offered) by race and ethnicity, though this warrants further study, as it contradicts much of the previous literature. Future research on employees’ perspectives, including a focus on real-world implementation and experiences of wGT users, is critical to understanding the perceived benefits and risks, thereby implementing best practices for future testing offerings through wGT or other similar testing avenues.

## Supplementary Information

ESM 1 (DOCX 209 kb)

## Data Availability

The survey data that support the findings of this study will be made available via ICPSR, housed at the University of Michigan.

## Declarations

### The INSIGHT @ Work Consortium

Elizabeth Charnysh, Betty Cohn, Nicole Crumpler, W. Gregory Feero, Rebecca Ferber, Veda N. Giri, Katherine Hendy, Amy Leader, Charles Lee, Debra Mathews, Sarah McCain, Alex McClellan, Anya E.R. Prince, J. Scott Roberts, Kerry Ryan, Kunal Sanghavi, Kayte Spector-Bagdady, Wendy R. Uhlmann, and Alyx Vogle.
