# Supplementary material for: Views and experiences regarding workplace genetic testing: findings from a national survey of U.S. employees
Source: J Community Genet. 2026 Mar 21;17(2):38. doi: 10.1007/s12687-025-00856-6 (PMC13004782; doi:10.1007/s12687-025-00856-6)
Supplement: Supplementary file 1 — (DOCX 209 kb) [file 12687_2025_856_MOESM1_ESM.docx]

**Journal Name:** *Journal of Community Genetics*

**TITLE:** Views and Experiences Regarding Workplace Genetic Testing: Findings from a National Survey of U.S. Employees

**AUTHORS:** Drew Blasco, PhD^1^, Sarah McCain, MPH^2^, Subhamoy Pal, PhD^3^, Wendy R. Uhlmann, MS, CGC^4^, Rebecca Ferber, MPH^5^, Kunal Sanghavi, CGC, MBBS^6^, Elizabeth Charnysh, MS, CGC^6^, Anya E.R. Prince, JD, MPP^7^, Charles Lee, PhD, FACMG^6^, J. Scott Roberts, PhD^2^, for The INSIGHT @ Work Consortium

**AFFILIATIONS:**

1. University of Nevada, Las Vegas, Department of Social and Behavioral Health, School of Public Health, Las Vegas, Nevada, USA
2. University of Michigan School of Public Health, Department of Health Behavior & Health Equity, Ann Arbor, Michigan, USA
3. University of Michigan School of Medicine, Department of Neurology, Ann Arbor, Michigan, USA
4. University of Michigan School of Medicine, Departments of Human Genetics and Internal Medicine, Ann Arbor, Michigan, USA
5. University of Michigan Medical School, Department of Internal Medicine, Ann Arbor, Michigan, USA
6. The Jackson Laboratory for Genomic Medicine, Farmington, Connecticut, USA
7. University of Iowa College of Law, Iowa City, Iowa, USA

**Corresponding Author’s Email:** jscottr@umich.edu

**SUPPLEMENTAL MATERIALS:**

**Supplemental Information 1 Dynata Survey Items:**

**Q11:** Many employers now have **workplace wellness activities**: promotions and programs aimed at supporting healthy behavior and improving health outcomes among employees. For example, an employer might offer smoking cessation or walking step programs, free blood pressure screening, or gym membership subsidies.

Does your employer offer any workplace wellness activities?

- Yes
- No
- Not sure

**Q11a:** If yes, have you participated in any of these workplace wellness activities?

- Yes
- No, but I may do so in the future
- No, and I do not plan to do so in the future

**Q12:** Many employers are now also offering **optional genetic testing programs**. These programs are offered in addition to typical healthcare benefits and might include testing to identify genetic risks for common health conditions such as cancer and heart disease.

Does your employer offer such a genetic testing program?

- Yes
- No
- Not sure

**Q12b:** If yes, did you participate in the genetic testing program?

- Yes
- No, but I may do so in the future
- No, and I do not plan to do so in the future

**Q12c:** If yes, what types of genetic testing were offered through this program?

- Testing for risk of cancer
- Testing for risk of heart disease
- Testing for risk of other medical conditions
- Testing to predict response to medications (pharmacogenomics)
- Carrier testing (to identify whether you have gene changes that increase future genetic risks to your biological children)
- Testing for genetic markers that predict responses to workplace toxin exposures (e.g., Beryllium, which can cause chronic beryllium disease)
- Fitness testing (genetic information to inform your exercise regimen)
- Nutrigenetic testing (to learn how one’s genes affect response to diet/foods)
- Ancestry testing (to learn what areas of the globe one’s ancestors came from)
- Testing for certain traits (e.g., alcohol flush reaction, cleft chin)
- Other
- I don’t know

**Q12f:** How was this genetic testing program offered?

- Testing through an onsite service provided by my employer
- Testing through an outside genetic testing company chosen by my employer
- Testing through my own primary care provider
- Testing through a genetics clinic
- Testing through a genetic testing company I chose on my own
- Other

**Q13:** If your employer offered a health-related genetic testing program, would you be interested in participating?

- Definitely yes
- Probably yes
- Not sure
- Probably not
- Definitely not

**Q14:** How interested would you be in the following types of genetic testing? (*Not at all; Somewhat; Very*)

- Genetic testing for risk of cancer
- Genetic testing for risk of heart disease
- Genetic testing for risk of Alzheimer’s disease
- Testing to predict response to medications (pharmacogenomics)
- Carrier testing (to identify whether you have gene changes that increase future genetic risks to your biological children)
- Testing for genetic markers that predict responses to workplace toxin exposures (e.g., Beryllium, which can cause chronic beryllium disease)
- Fitness testing (genetic information to inform your exercise regimen)
- Nutrigenetic testing (to learn how one’s genes affect response to diet/foods)
- Ancestry testing (to learn what areas of the globe one’s ancestors came from)
- Testing for certain traits (e.g., alcohol flush reaction, cleft chin)

**Q15:** Below are some reasons why people might be interested in genetic testing in the workplace.

How important were the following in your decision about whether or not to undergo genetic testing through your employer? (*Not at all; Somewhat; Very; Extremely*)

- Results might inform my medical care
- Results might assist me in planning for the future
- Results might inform my health behaviors and lifestyle choices
- Results might be important for my family members to know about
- Results might help me to learn more about my genetics because I lack information about my family history

**Q15a:** What do you view as the most important reason to participate in testing?

- - Results might inform my medical care
  - Results might assist me in planning for the future
  - Results might inform my health behaviors and lifestyle choices
  - Results might be important for my family members to know about
  - Results might help me to learn more about my genetics because I lack information about my family history

**Q16:** Below are some reasons why people might NOT be interested in genetic testing in the workplace.

How important were the following factors in your decision whether or not to undergo genetic testing through your employer? (*Not at all; Somewhat; Very; Extremely*)

- The results might not be useful or informative
- The results might make me worried or anxious
- The results might be confusing or difficult to understand
- I would be concerned about my employer having access to my personal genetic information
- I would be concerned that results might affect my ability to obtain insurance and/or the cost of my insurance premiums
- I would be concerned that results might affect my ability to get or keep a job

**Q16a:** What do you view as the **most important** reason NOT to participate in testing?

- - The results might not be useful or informative
  - The results might make me worried or anxious
  - The results might be confusing or difficult to understand
  - I would be concerned about my employer having access to my personal genetic information
  - I would be concerned that results might affect my ability to obtain insurance and/or the cost of my insurance premiums
  - I would be concerned that results might affect my ability to get or keep a job

**Q17:** There are several different ways in which employers could offer genetic testing in the workplace, some of which are listed below. **Please indicate which of the following options you would consider**. (Check all that apply) Assume that under each option, your employer would pay all costs associated with the testing.

- Testing through an onsite service provided by my employer
- Testing through an outside genetic testing company chosen by my employer
- Testing through my own primary care provider
- Testing through a genetics clinic
- Testing through a genetic testing company I chose on my own

**Q17a:** Which one would you prefer most?

- - Testing through an onsite service provided by my employer
  - Testing through an outside genetic testing company chosen by my employer
  - Testing through my own primary care provider
  - Testing through a genetics clinic
  - Testing through a genetic testing company I chose on my own

**Q18:** If an employer were to offer genetic testing in the workplace, how important would it be to include the following features? (*Not at all; Somewhat; Very; Extremely*)

- Assurance that employees’ test results would not be shared with supervisors or coworkers
- Assurance that employees’ test results would not impact their health insurance
- Genetic counseling services to help interpret results
- Testing done at the work site for employee convenience
- Service provided at no cost to employees
- Information provided on laws protecting against genetic discrimination
- Data would not be shared without employee permission, even if de-identified
- Other

**Q25:** Now, we’d like to ask you about your experiences with other kinds of genetic testing, beyond those that may have been offered through your employer. Have you ever had genetic testing offered in the following ways? (Select all that apply)

- Yes, through a healthcare provider
- Yes, through a direct-to-consumer service (e.g., 23andMe, AncestryDNA)
- Yes, through a research study
- No

**Q27:** How familiar are you with genetic testing?

- Not very familiar
- Somewhat familiar
- Very familiar

**Supplemental Fig. 1** Employer Offered wGT, Uptake Among Those Offered, and Interest Among Those Reporting Their Employer Did Not Offer/Were Unsure if Their Employer Offered wGT


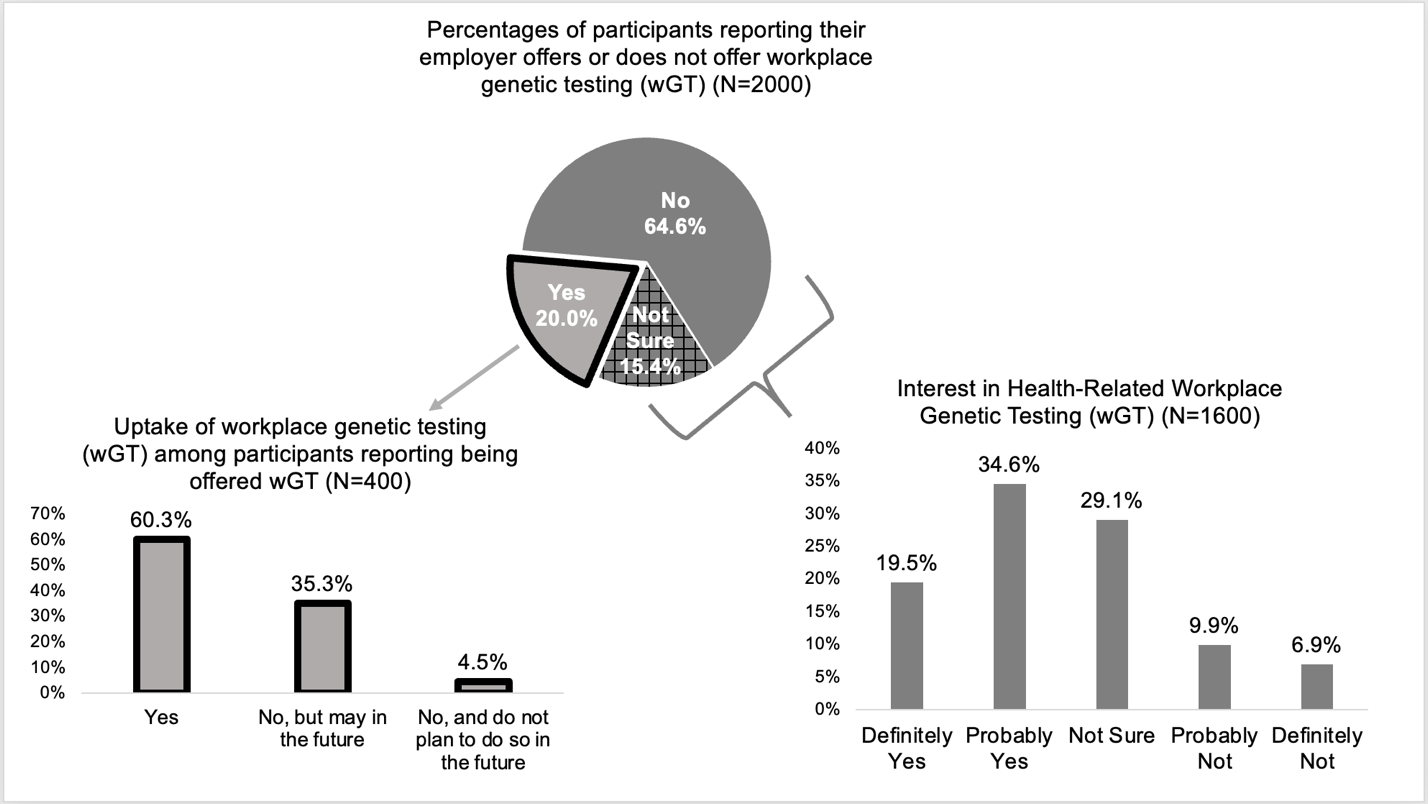


Note: Participants who indicated their employer *did not offer wGT* or *were not sure if their employer offered wGT* were asked about their interest in participating in health-related wGT should their employer offer it.
